# Circadian Disorganization Alters Intestinal Microbiota

**DOI:** 10.1371/journal.pone.0097500

**Published:** 2014-05-21

**Authors:** Robin M. Voigt, Christopher B. Forsyth, Stefan J. Green, Ece Mutlu, Phillip Engen, Martha H. Vitaterna, Fred W. Turek, Ali Keshavarzian

**Affiliations:** 1 Department of Internal Medicine, Division of Gastroenterology, Rush University Medical Center, Chicago, Illinois, United States of America; 2 DNA Services Facility, Research Resources Center, University of Illinois at Chicago, Chicago, Illinois, United States of America; 3 Department of Biological Sciences, University of Illinois at Chicago, Chicago, Illinois, United States of America; 4 Center for Sleep & Circadian Biology, Northwestern University, Evanston, Illinois, United States of America; 5 Department of Biochemistry, Rush University Medical Center, Chicago, Illinois, United States of America; 6 Department of Pharmacology, Rush University Medical Center, Chicago, Illinois, United States of America; 7 Department of Physiology, Rush University Medical Center, Chicago, Illinois, United States of America; 8 Division of Pharmacology, Utrecht Institute for Pharmaceutical Sciences, Utrecht University, Utrecht, The Netherlands; McGill University, Canada

## Abstract

Intestinal dysbiosis and circadian rhythm disruption are associated with similar diseases including obesity, metabolic syndrome, and inflammatory bowel disease. Despite the overlap, the potential relationship between circadian disorganization and dysbiosis is unknown; thus, in the present study, a model of chronic circadian disruption was used to determine the impact on the intestinal microbiome. Male C57BL/6J mice underwent once weekly phase reversals of the light:dark cycle (i.e., circadian rhythm disrupted mice) to determine the impact of circadian rhythm disruption on the intestinal microbiome and were fed either standard chow or a high-fat, high-sugar diet to determine how diet influences circadian disruption-induced effects on the microbiome. Weekly phase reversals of the light:dark (LD) cycle did not alter the microbiome in mice fed standard chow; however, mice fed a high-fat, high-sugar diet in conjunction with phase shifts in the light:dark cycle had significantly altered microbiota. While it is yet to be established if some of the adverse effects associated with circadian disorganization in humans (e.g., shift workers, travelers moving across time zones, and in individuals with social jet lag) are mediated by dysbiosis, the current study demonstrates that circadian disorganization can impact the intestinal microbiota which may have implications for inflammatory diseases.

## Introduction

High throughput DNA/RNA-based technology has led to a revolution in our understanding of the complexity and diversity of the microbiota communities that inhabit many parts of the human body, including those in the intestine. It is now recognized that the number of microbial cells in the gastrointestinal tract outnumber the total human cells in the body, with an estimated 10^14^ cells, most of them being bacteria [1], [2]. Microbiota community composition and diversity are highly malleable and sensitive to changes in the host including environmental factors such as diet, and disruption of normal bacterial communities in the intestine (i.e., dysbiosis) has been linked to several chronic inflammatory diseases and pathological conditions [3], [4]. It has been known for over 40 years that the suprachiasmatic nucleus (SCN) in the mammalian hypothalamus contains the central circadian clock that regulates a myriad of cellular, physiological, and behavioral 24-hr rhythms. The discovery of circadian clock genes in mammals has led to a revolution in our understanding of how most, if not all, cells in the body contain the molecular machinery necessary to generate circadian rhythms. In fact, the genes and proteins comprising the core molecular clock regulate the timing of the expression of about 10% of all transcripts produced in any given tissue or organ [5]. Recently, a variety of human diseases, typically those associated with chronic inflammation (e.g., obesity, inflammatory bowel disease (IBD), cancer, and neurological disorders), have been found to occur in conjunction with either disruption of normal microbiota communities or circadian organization. It is not known if a common mechanism mediates the effects of disrupted microbiota or circadian rhythms, nor how disrupting intestinal microbiota or the circadian rhythms of the host might impact the other.

The current study stemmed from our previous publication demonstrating that 12 weeks of circadian rhythm disruption promoted intestinal hyperpermeability, an effect that was exacerbated when mice were fed a high-fat, high-sugar diet for 10 weeks. This effect seems to be, at least in part, due to effects on the tight junction protein occludin [6]. In an effort to better understand the mechanism by which circadian rhythm disruption may influence intestinal permeability, stool collected from mice in the previous study was analyzed. As a first step to elucidate how the intestinal microbiota and circadian systems might interact, we examined the impact of chronic circadian rhythm disruption on the intestinal microbiota composition of mice fed a standard laboratory chow diet or a high-fat, high-sugar diet. Since we have previously demonstrated that repeated phase shifts in the light:dark cycle can disrupt intestinal barrier function [6] and render the colon more susceptible to injury in an experimentally induced model of ulcerative colitis [7], and because changes in microbiota are known to occur during both pathological conditions [3], [8], [9], we used this phase-shift model to determine if circadian rhythm disruption impacted intestinal microbiota composition.

## Methods

### Ethics Statement

All mice were housed and handled in accordance with federal animal welfare guidelines and in compliance with the Public Health Service Policy on Humane Care and Use of Laboratory Animals (2002) and the Guide for the Use and Care of Laboratory Animals (8^th^ Edition). All experiments were reviewed and approved prior to being conducted by the Institutional Animal Care and Use Committee at Rush University Medical Center (Animal Study Protocol #10-083).

### Mice and Housing

Studies were conducted at Rush University Medical Center in accordance with Federal animal welfare guidelines and in compliance with the Public Health Service Policy on Humane Care and Use of Laboratory Animals and the Guide for the Use and Care of Laboratory Animals. All experimental protocols were reviewed and approved by the Institutional Animal Care and Use Committee of Rush University Medical Center. Thirty-three young adult (6–8 week), male wild-type C57BL/6J mice obtained from Jackson Laboratory (Bar Harbor, ME) were housed individually in cages stored within ventilated, light-tight cabinets. Mice were acclimated to the facility for one week prior to initiating the experiment. Locomotor activity, food intake, and body weight were measured throughout the experiment.

### Circadian Manipulation

**Figure 1:**
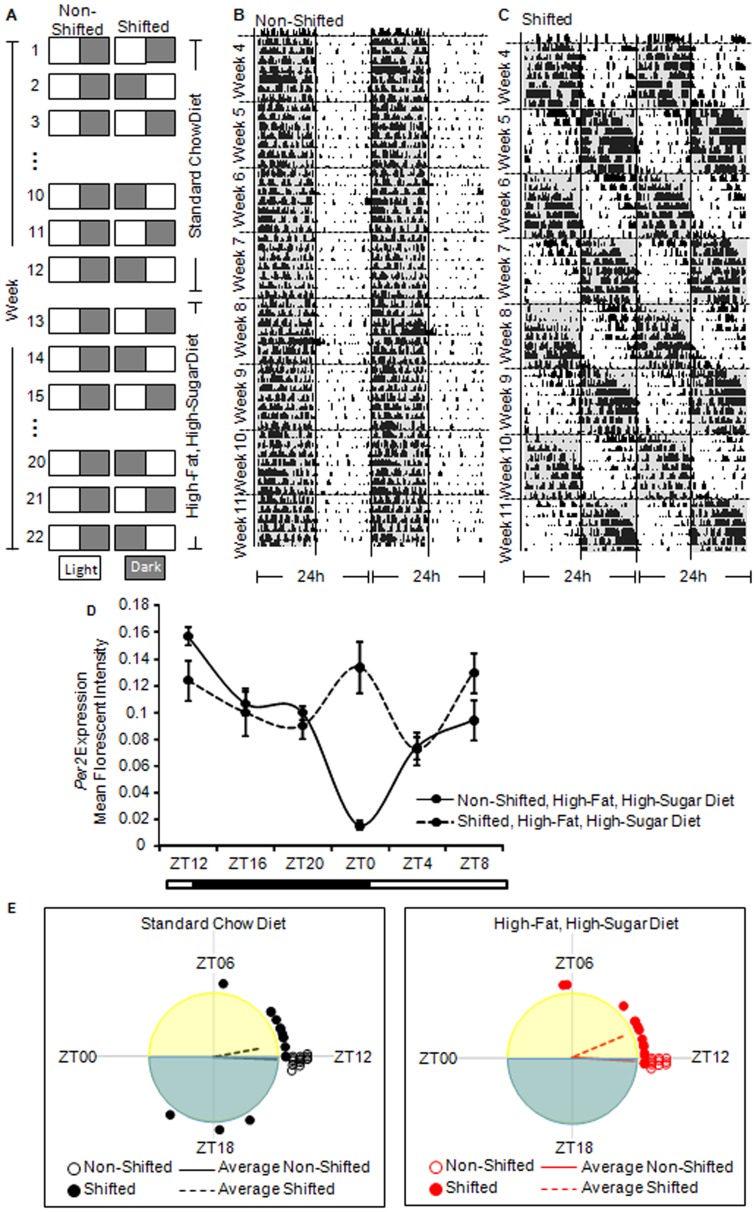
Protocol and timeline for circadian disruption, dietary changes, and stool collection. A. Non-shifted mice were kept on a constant light:dark schedule for the entirety of the experiment whereas Shifted mice underwent once weekly light:dark inversion. Mice were maintained on a standard chow diet for the first 12 weeks of the study followed by a stool collection at the end of week 12. Mice were subsequently placed on a high-fat, high-sugar diet for 10 weeks and stool was collected at the end Week 22. Representative behavioral activity recording for a Non-shifted (B) and Shifted mouse (C). Activity is represented as dark areas in the actogram. These data demonstrate that non-shifted mice have stable behavioral patterns while the shifted mice have disrupted behavioral patterns. D. Expression of *Per2* mRNA in the proximal colon one day after the week 22 stool collection demonstrates altered circadian expression in the intestine following the 22 weeks of phase shifting. Significant effect of light condition, (F_(1,58)_ = 5.84, p = 0.02), Zeitgeber Time (ZT) (F_(5,58)_ = 8.38, p<0.0001), and light condition x ZT interaction (F_(5,58)_ = 9.67, p = <0.0001). E. Activity onset phase distribution on the day of stool collection for standard chow-fed and (left) and high-fat, high-sugar diet (right). Individual mouse onset times relative to the light:dark cycle are depicted by the circles, and vector means depicted by the lines. There were no significant differences between the mean phase angle of entrainment between the shifted and non-shifted mice at the time of stool sample collection, but shifted mice exhibited a greater dispersal of phases in both diets (p<0.05).

The circadian manipulation protocol was conducted as described in our previous publication [6]. The phase shift in the light:dark cycle every week (Figure 1A) is intended to disrupt the normal synchrony of the circadian clock system to the entraining light:dark cycle in the same way that flying rapidly across time zones (for mice in this study, 12 time zones) leads to jet lag in humans. In brief, following the acclimation period, the mice were randomized into one of two groups: (1) the non-shifted group, maintained on a constant light:dark cycle or (2) the shifted group, underwent weekly reversals of the light:dark cycle. These circadian protocols were maintained for the duration of the experiment (i.e., 22 weeks). Twelve weeks of circadian rhythm disruption is sufficient to promote intestinal hyperpermeability [6] and exacerbate dextran sodium sulfate (DSS)-induced effects in a mouse model of ulcerative colits [10] and 22 weeks of circadian rhythm disruption is sufficient to markedly increase alcohol-induced intestinal hyperpermeability [6]; thus, the 22 week study period parallels studies we have already established negatively impact health. Locomotor activity records of a representative non-shifted (Figure 1B) and shifted (Figure 1C) mouse indicate the stable entrainment (non-shifted) or the repeated re-entrainment/disturbance (shifted) of the circadian locomotor behavioral rhythm. In this example (Figure 1C), it took an average of six days for the mouse to re-entrain to the weekly 12-hour phase shift, that is, it was phase delaying about two hours each day. This phenomenon can give the appearance that the mouse was free running with a period of approximately 26 hours. The free running period of C57BL/6J mice is approximately 23.7 hours; we have never observed a period greater than 24.3 hours in C57BL/6J mice unless the mouse is carrying a genetic mutation in a core clock gene. Figure 1D depicts altered RNA expression of the circadian gene *Per2* in the proximal colon following 22 weeks of 12 hour light:dark inversions supporting that our chronic shifting paradigm disrupted circadian rhythmicity not only behaviorally but also circadian gene expression in the intestine.

In our study, individual mice took from four to seven days to re-entrain to the light:dark cycle. At the time when tissue samples were collected (seven days after the last phase shift) the phase angles between activity onset and lights off were significantly clustered (p<0.0001, Rayleigh test) with a mean of 1.31±0.53 h, indicative of their re-entrainment (Figure 1E). While the distributions of phases were different between non-shifted and shifted mice at the times of stool collection (Figure 1E), these distributions were attributable to differences in variance (or concentration parameter) rather than mean phase [11]. Although no significant differences in mean phase angle were detected, we still examined the potential impact of entrainment on microbiota composition (described in Results).

### Feeding Protocol

The feeding protocol was conducted as described in our previous publication [6]. Briefly, all mice were maintained on standard rodent chow (Harlan Teklad Global 18% Protein Rodent Diet; 18.6% protein, 44.2% carbohydrate, 6.2% fat, 3.5% crude fiber) for the first 12 weeks of the study. After this time, mice were switched to a high-fat, high-sugar diet (36% protein, 29% carbohydrate (dextrose), and 35% fat) that contained: mineral mix, vitamin mix, choline bitartrate, DL-methionine, lactalalbumin, xanthan gum, dextrose (Dyets, Bethlehem, PA), fish oil from menhaden (Sigma, St. Louis, MO), and Hersey's chocolate syrup. Fish oil was selected as the source of fat based on data demonstrating that unsaturated fat (i.e., fish oil) promotes the development of endotoxemia and alcoholic steatohepatitis by increasing cytochrome P450 2E1 induction and lipid peroxidation [12], [13]. The diet was prepared fresh daily and supplied to mice in specialized graduated sipper tubes (Bio-Serv, Frenchtown, NJ), for daily monitoring of food intake.

### Tissue Collection & RNA analysis

The tissue collection protocol was conducted as described in our previous publication [6]. Briefly, at the end of the experiment, mice were euthanized by conscious decapitation. Proximal colon was harvested and placed into RNALater (Qiagen, Valencia, CA) and frozen. Tissues were stored at −80°C until use. Tissues were collected every four hours across the diurnal cycle, starting at ZT0 (i.e., ZT0, ZT4, ZT8, ZT12, ZT16, and ZT20). Clock mRNA was measured in proximal colon samples prepared as tissue homogenates using Affymetrix lysis buffer and processed according to manufacturer's instructions. mRNA levels were determined using a Luminex platform-based custom multiplex bead array (Affymetrix, Inc., Santa Clara, CA). Expression was normalized to the housekeeping gene RPLPO. Data are expressed as mean fluorescent intensity (MFI).

#### Stool Collection

Spontaneously voided stool pellets were collected at two times: (1) after 12 weeks on the standard chow diet (Week 12) and (2) after 10 weeks on the high-fat, high-sugar diet (Figure 1A). These time points were selected as they corresponded to times when intestinal permeability was measured, a phenomenon that may be directly or indirectly influenced by intestinal microbiota composition [6]. Mice were placed into a metabolic cage for five hours beginning at lights on (ZT0-ZT5, with ZT0 = lights on) and stool produced during this five hour period was collected and stored at −80°C.

### Microbial Community Analysis

Genomic DNA was obtained using an extraction kit according to manufacturer's instructions (MP Biomedicals FastDNA SPIN Kit for Soil, #116560200). Genomic DNA samples were sent to Research and Testing Laboratory (Lubbock, TX) for amplification and sequencing of fragments of bacterial small subunit (SSU or 16S) ribosomal RNA (rRNA) genes. Briefly, PCR amplification was performed using the primers Gray28F and Gray519r [14] and sequencing reactions were performed on a Roche 454 FLX instrument (Roche, Indianapolis, IN) with Titanium reagents, titanium procedures, a one-step PCR, and a mixture of Hot Start and Hot Star high-fidelity Taq polymerases. After sequencing, all failed sequence reads and low-quality sequences were removed, and sequences were depleted of any nonbacterial ribosome sequences and chimeras using custom software [15], as described previously [16]. Clustering sequences into taxonomic groups from species to phylum was performed with the software package USEARCH [17], and taxonomic affiliation of sequences was performed by query of representative sequences against a curated 16S rRNA gene database, as described previously (*e.g.* Andreotti et al. 2011).

For determination of diversity, raw sequences were quality filtered at a Q20 level within the software package CLC genomics workbench (CLC bio, Denmark), and sequences shorter than 275 bases were removed. Subsequently, each sample set was randomly sub-sampled to the same number of sequences (800 sequences/sample, ∼75% of the smallest library) to allow for direct comparison of calculated diversity indices. Sequences from each sample were pooled and processed through the Ribosomal Database Project's (RDP) Pyrosequencing pipeline [18] (http://pyro.cme.msu.edu/). Briefly, all sequences were aligned, and clustered using a 0.03 similarity threshold for complete linkage clustering. The number of sequences from each sample cluster was identified and a biological observation matrix (BIOM) was generated [19]. The BIOM was analyzed using the software package Primer6 (v6.1.15; PrimerE). Univariate diversity analysis (i.e., Shannon index) was implemented using the DIVERSE function in Primer6.

Species-Taxonomic affiliation of clustered sequence data were determined as described above, and these data were used to generate multiple BIOMs at multiple taxonomic levels, including species, genus, and family. These BIOMs were subsequently used for analysis of microbial community structure and statistical analyses. For hypothesis testing, species-level data organized in a BIOM were pre-treated to standardize taxon abundance within each sample, and the data were subsequently square-root transformed to down-weight the impact of high abundance species. The transformed data were then used to generate a pair-wise resemblance matrix based on Bray-Curtis similarity (Primer6) that was analyzed using hierarchical clustering (group average) and non-metric multi-dimensional scaling (NMDS). Analysis of similarities (ANOSIM) was used to determine statistically significant changes in microbial community structure in *a priori* defined sample grouping. To do so, a test statistic “R” was calculated from the average distance of all members of a group and contrasted with average distances between replicates of different groups. The R statistic is calculated so that R = 1 if all replicates within groups are more similar than any replicates from different samples and R values approaching zero indicate dissimilarity. To determine significance, the R statistic was calculated under permutations of the sample labels, and the measured R value was compared to the permutation distribution [20]. Similarity percentage for each group (5-10/group) was independently analyzed. Community dissimilarity between treatment groups was calculated using the ‘similarity percentages' (SIMPER) function within Primer6, employing resemblance based on Bray Curtis similarity.

Treatment effects were also analyzed at higher taxonomic level, employing a BIOM generated from family-level clustered sequence data. Principal coordinates analysis (PCA) was performed within the software package Canoco (v5) [21]. Data were standardized by sample, and log-transformed prior to PCA.

### Data Access

The 16S rRNA gene amplicon sequence data from this study have been submitted to the NCBI Sequence ReadArchive (http://www.ncbi.nlm.nih.gov/Traces/sra/sra.cgi) under accession number SRP029435.

## Results

Figure 1 depicts the treatment protocol (Figure 1A), locomotor activity record of a representative non-shifted and shifted mouse to indicate the stable entrainment (non-shifted, Figure 1B) or the repeated disturbance of the circadian locomotor behavioral rhythm (shifted, Figure 1C), and expression of the circadian gene *Per2*in the proximal colon to verify disruption of circadian rhythms (Figure 1D). There was a significant effect of light condition (p = 0.020), Zeitgeber time (p<0.0001), as well as a light condition x time interaction (p<0.0001) on *Per2* levels (two-way ANOVA). A total of 36 stool samples were collected during weeks 12 and 22, and 33 were used for the analysis (three samples in the circadian disrupted, high-fat, high-sugar diet were below the detection level of DNA necessary for analysis). Genomic DNA was extracted from stool pellets and was used as templates for PCR amplification of partial bacterial small subunit (SSU or 16S) ribosomal RNA (rRNA) genes [14]. In total, 33 samples belonging to four treatment groups were analyzed. A total of 113,475 sequences were recovered (average = 3,439/sample; range 1,061–6,601).

**Figure 2:**
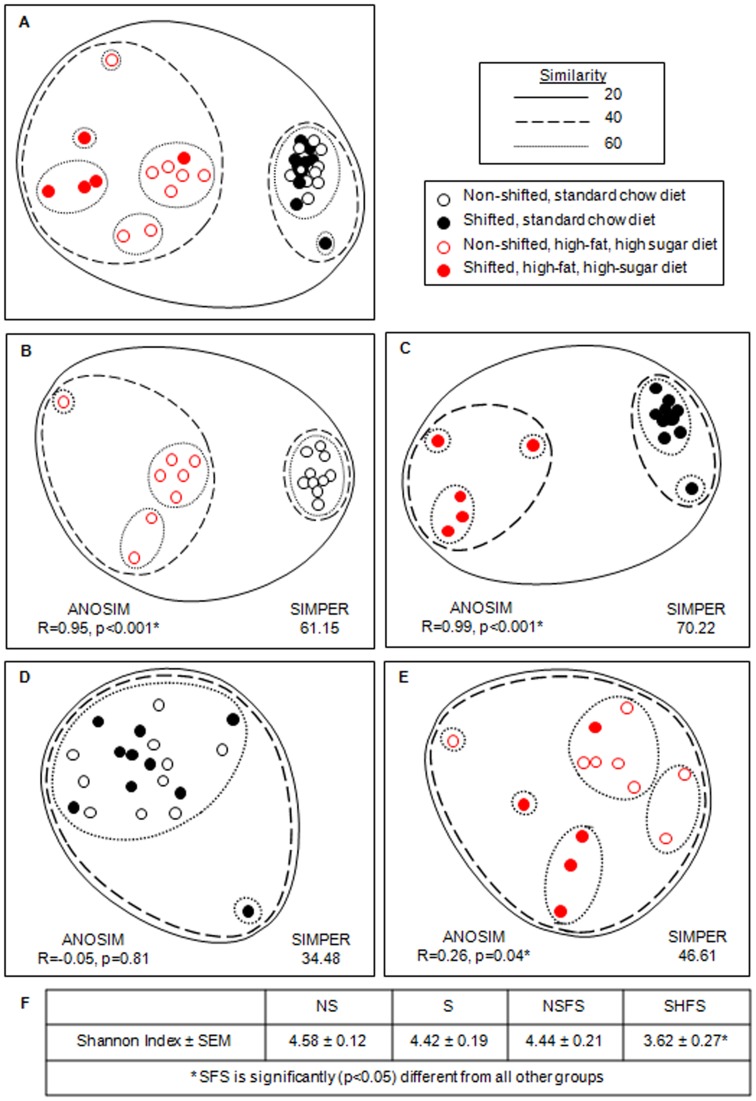
Effect of diet and circadian rhythm disruption on mouse gut microbial communities. The non-metric multidimensional scaling (NMDS) plot demonstrates the effect of treatments on the overall mouse fecal microbial community structure, as assessed by bacterial small subunit ribosomal RNA gene amplicon sequencing . The NMDS plot is based on sample-standardized and square-root transformed abundance data. The NMDS plot and the hierarchical cluster overlay are based on a resemblance matrix calculated using S17 Bray-Curtis similarity. 2D stress values ranged from 0.04 to 0.17. ANOSIM: Analysis of similarities. SIMPER: Similarity percentages.

The treatment effects on the overall microbial community structure are shown using non-metric multidimensional scaling (Figure 2A). Maintenance of mice on a high-fat, high-sugar diet for 10 weeks resulted in a significant shift in the intestinal microbiota composition (Figure 2A; ANOSIM: R = 0.96, p = 0.01 (9999 permutations); SIMPER: 64.49), an effect that was true for non-shifted (Figure 2B; ANISOM: R = 0.95, p = 0.00002; SIMPER: 61.15) and as well as circadian rhythm disrupted mice (Figure 2C; ANISOM: R = 0.99, p = 0.0003; SIMPER: 70.22). Chronic circadian rhythm disruption did not have an effect on the microbiota of mice fed standard chow (Figure 2D; ANISOM: R = −0.05, p = 0.81; SIMPER: 34.48); however, statistically significant differences were observed when high-fat, high-sugar diet-fed mice underwent weekly phase shifts in the light:dark cycle (Figure 2E; ANISOM: R = 0.26, p = 0.04; SIMPER: 46.61). As would be expected, the high-fat, high-sugar diet had an overt effect on microbiota composition, regardless of circadian status. Circadian rhythm disruption also altered the gut microbial community composition, but this effect was noted only when mice were fed the high-fat, high-sugar diet. To account for any potential effects of differences in phase at the time of sample collection among shifted mice on stool microbiota composition, we compared stool collected from mice that had fully re-entrained (i.e., activity onset within one hour of lights off) at the time of stool collection to stool collected from mice that had not yet fully re-entrained to the light:dark cycle. Within this small sample size, significant effects of entrainment were not observed on stool microbial composition in either the standard chow diet (re-entrained vs. nearly entrained, p = 0.93) or the high-fat, high-sugar diet (re-entrained vs. nearly entrained, p = 1.00). These data demonstrate that circadian disruption can significantly impact intestinal microbiota community structure when combined with a high-fat, high-sugar diet, and that this impact is not attributable to the circadian phase when samples were collected.

Microbial diversity was assessed in rarefied libraries, and no significant difference in number of taxa (i.e., OTU_97_), Margalef richness, Pieoulus evenness, or Shannon index were observed in non-shifted, standard chow fed mice or in shifted, standard chow-fed mice or non-shifted high-fat, high-sugar diet-fed mice. In contrast, microbial diversity was significantly reduced in the shifted mice fed the high-fat, high-sugar diet (Average Shannon indices: non-shifted, stand-chow fed mice: 4.58±0.12; shifted standard chow-fed mice: 4.42±0.19; non-shifted high-fat, high-sugar diet-fed mice: 4.44±0.21; shifted, high-fat, high-sugar diet-fed mice: 3.62±0.27; one-way ANOVA, p = 0.05). These data demonstrate that the combination of the high-fat, high-sugar diet and circadian rhythm disruption significantly impacted diversity.

**Figure 3:**
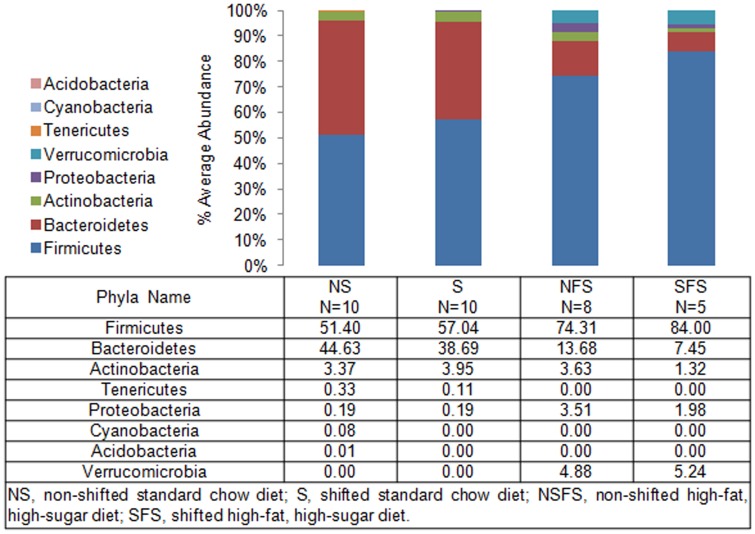
Mouse gut microbial community structure at the phylum level. The bar graph graphically represents the average relative abundance of classified bacteria SSU rRNA gene amplicons belonging to the most abundant phyla.

**Figure 4:**
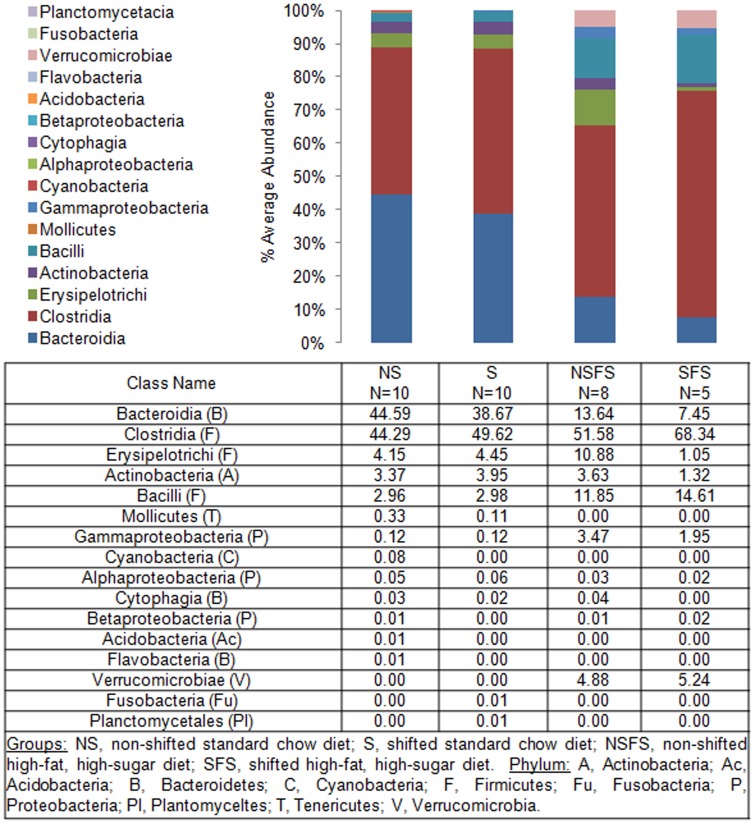
Mouse gut microbial community structure at the class level. The bar graph graphically represents the average relative abundance of classified bacteria SSU rRNA gene amplicons belonging to the most abundant taxon at the class level.

**Figure 5:**
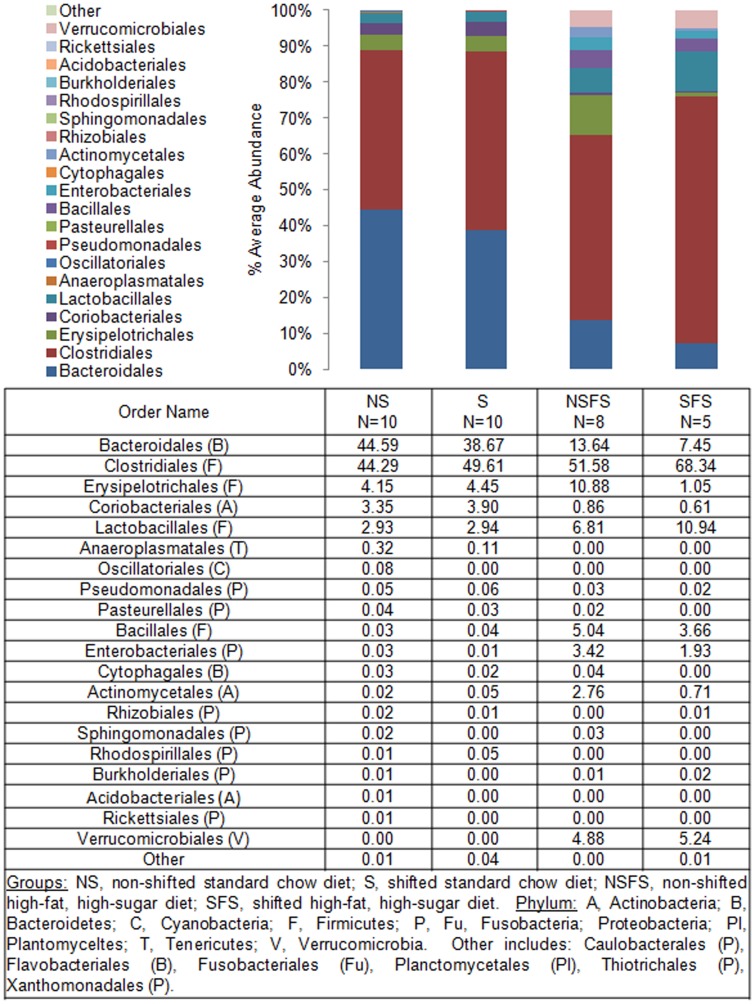
Mouse gut microbial community structure at the order level. The bar graph graphically represents the average relative abundance of classified bacteria SSU rRNA gene amplicons belonging to the most abundant taxon at the order level.

**Figure 6:**
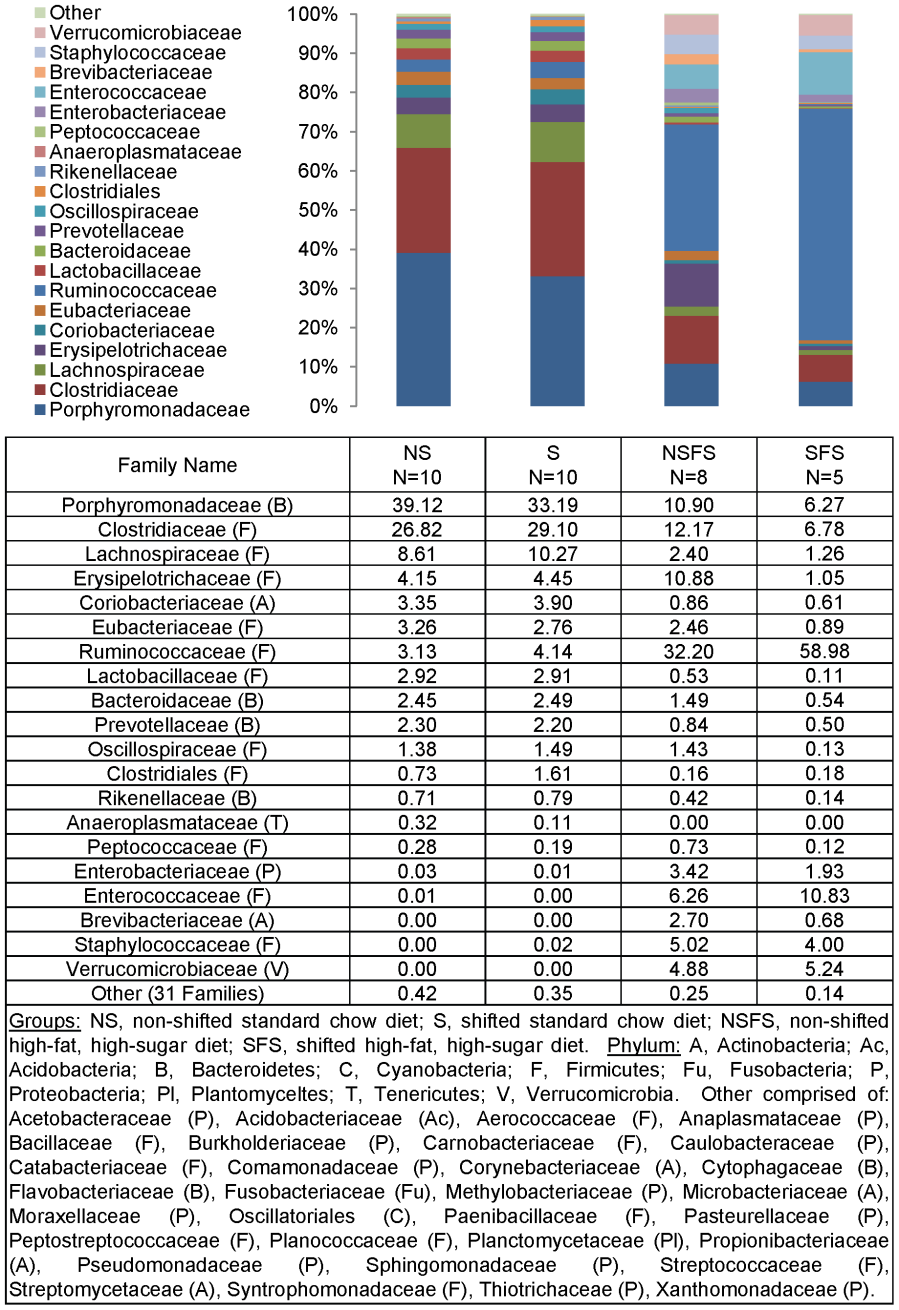
Mouse gut microbial community structure at the family level. The bar graph graphically represents the average relative abundance of classified bacteria SSU rRNA gene amplicons belonging to the most abundant taxon at the family level.

To define shifts in microbiota community composition associated with each experimental group, average taxonomic group abundance was determined at various taxon levels (phylum, class, order, family, and genus) and statistical differences were identified using an ANOVA followed by *a priori* established pair wise comparisons (i.e., Student's *t*-test) to test specific hypotheses. Alterations in microbiota community composition were observed at multiple taxonomic levels. Average abundance of the dominant phylum is shown in Figure 3 with statistical outcomes listed in Table 1. Statistical outcomes for class, order, family, and genus taxon comparisons are listed in Tables 2–5 with average abundance of each taxon depicted in Figures 4–6 and genus listed in Table 6. Intestinal microbial communities were dramatically impacted by the high-fat, high-sugar diet, and this was manifested as significant increases in the relative abundance of bacteria from the phyla Firmicutes, Proteobacteria, and Verrumicrobia, with a concomitant reduction in the average relative abundance of bacteria from the phylum Bacteroidetes (Figure 3, Table 1). Significant circadian rhythm-induced changes were detected at the family and genus levels, but not at the phylum level (Tables 1–5). Bacteria from the phylum Firmicutes were most strongly impacted by the combination of the high-fat, high-sugar diet and circadian rhythm disruption, and both increases and decreases in the relative abundance of taxa were observed (e.g., *Desulfosporosinus* and *Desulfotomaculum* decreased and *Ruminococcus* and *Sporosarcina* increased in relative abundance). Thus, shifts in community composition were observed as a consequence of a high-fat, high-sugar diet, an effect that was further augmented by circadian rhythm disruption.

**Table 1:** Significant between group comparisons by taxon level.

Taxon Name	Diet Effect		Circadian Effect		ANOVA
	NS v NSFS	S v SFS	NS v S	NSFS v SFS	
Phylum					
Bacteroidetes	<0.00* (−)	<0.00* (−)	–	–	<0.00*
Firmicutes	<0.01* (+)	0.00* (+)	–	–	<0.00*
Proteobacteria	<0.00* (+)	0.03* (+)	–	–	<0.00*
Verrucomicrobia	0.01* (+)	0.02* (+)	–	–	<0.00*

P values indicated. Non-significant between group differences observed for: Acidobacteria, Actinobacteria, Cyanobacteria, Fusobacteria, Planctomycetes, Tenericutes. Groups: NS, non-shifted standard chow diet; S, shifted standard chow diet; NSFS, non-shifted high-fat, high-sugar diet; SFS, shifted high-fat, high-sugar diet. *, significant between group differences; –, not significant; (+)/(-), indicates direction of the change.

**Table 2:** Significant between group comparisons by taxon level.

Taxon Name	Diet Effect		Circadian Effect		ANOVA
	NS v NSFS	S v SFS	NS v S	NSFS v SFS	
Class					
Bacteroidia (B)	<0.00* (-)	<0.00* (-)	–	–	<0.00*
Gammaproteobacteria (P)	<0.00* (+)	0.03* (+)	–	–	<0.00*
Verrucomicrobiae (V)	0.01* (+)	0.02* (+)	–	–	<0.00*

P values indicated. Non-significant between group differences observed for: Acidobacteria (Ac), Actinobacteria (A), Alphaproteobacteria (P), Bacilli (F), Betaproteobacteria (P), Clostridia (F), Cyanobacteria (C), Cytophagia (B), Erysipelotrichi (F), Flavobacteria (B), Fusobacteria (Fu), Mollicutes (T), Planctomycetia (Pl). Groups: NS, non-shifted standard chow diet; S, shifted standard chow diet; NSFS, non-shifted high-fat, high-sugar diet; SFS, shifted high-fat, high-sugar diet. Phylum: A, Actinobacteria; Ac, Acidobacteria; B, Bacteroidetes; C, Cyanobacteria; F, Firmicutes; Fu, Fusobacteria; P, Proteobacteria; Pl, Plantomycetes; T, Tenericutes; V, Verrucomicrobia. *, significant between group differences; –, not significant; (+)/(-), indicates direction of the change.

**Table 3:** Significant between group comparisons by taxon level.

Taxon Name	Diet Effect		Circadian Effect		ANOVA
	NS v NSFS	S v SFS	NS v S	NSFS v SFS	
Order					
Bacteroidales (B)	<0.00* (-)	<0.00* (-)	–	–	<0.00*
Enterobacteriales (P)	<0.00* (+)	0.02* (+)	–	–	<0.00*
Verrucomicrobiales (V)	0.01* (+)	0.02* (+)	–	–	<0.00*

P values indicated. Non-significant between group differences observed for: Acholeplasmatales (T), Acidobacteriales (Ac), Actinomycetales (A), Anaeroplasmatales (T), Bacillales (F), Burkholderiales (P), Caulobacterales (P), Clostridiales (F), Coriobacteriales (A), Cytophagales (B), Erysipelotrichales (F), Flavobacteriales (B), Fusobacteriales (Fu), Lactobacillales (F), Oscillatoriales (C), Pasteurellales (P), Planctomycetales (Pl), Pseudomonadales (P), Rhizobiales (P), Rhodospirillales (P), Rickettsiales (P), Sphingomonadales (P), Thermoanaerobacterales (F), Thiotrichales (P), Xanthomonadales (P). Groups: NS, non-shifted standard chow diet; S, shifted standard chow diet; NSFS, non-shifted high-fat, high-sugar diet; SFS, shifted high-fat, high-sugar diet. Phylum: A, Actinobacteria; Ac, Acidobacteria; B, Bacteroidetes; C, Cyanobacteria; F, Firmicutes; Fu, Fusobacteria; P, Proteobacteria; Pl, Plantomycetes; T, Tenericutes; V, Verrucomicrobia. *, significant between group differences; –, not significant; (+)/(-), indicates direction of the change.

**Table 4:** Significant between group comparisons by taxon level.

Taxon Name	Diet Effect		Circadian Effect		ANOVA
	NS v NSFS	S v SFS	NS v S	NSFS v SFS	
Family					
Bacteroidaceae (B)	–	<0.00* (−)	–	–	<0.00*
Porphyromonadaceae (B)	<0.00* (−)	<0.00* (−)	–	–	<0.00*
Prevotellaceae (B)	<0.00* (−)	0.00* (−)	–	–	<0.00*
Rikenellaceae (B)	–	<0.01* (−)	–	–	<0.00*
Catabacteriaceae (F)	0.00* (−)	–	0.02* (-)	–	<0.00*
Clostridiaceae (F)	<0.00* (−)	<0.00* (−)	–	–	<0.00*
Enterococcaceae (F)	–	–	–	–	0.03*
Lachnospiraceae (F)	<0.04* (−)	<0.01* (−)	–	–	<0.00*
Lactobacillaceae (F)	–	–	–	–	0.03*
Peptococcaceae (F)	0.02* (+)	–	–	0.01* (−)	<0.00*
Planococcaceae (F)	–	0.01* (+)	–	0.03* (+)	0.01*
Ruminococcaceae (F)	<0.00* (+)	0.00* (+)	–	0.01* (+)	<0.00*
Streptococcaceae (F)	–	–	–	–	0.04*
Enterobacteriaceae (P)	<0.00* (+)	0.02* (+)	–	–	<0.00*
Verrucomicrobiaceae (V)	0.01* (+)	0.02* (+)	–	–	<0.00*

P values indicated. Non-significant between group differences observed for: Acetobacteraceae (P), Acholeplasmataceae (T), Acidobacteriaceae (Ac), Aerococcaceae (F), Anaplasmataceae (P), Anaeroplasmataceae (T), Bacillaceae (F), Bradyrhizobiaceae (P), Brevibacteriaceae (A), Burkholderiaceae (P), Carnobacteriaceae (F), Caulobacteraceae (P), Clostridiales (F), Comamonadaceae (P), Coriobacteriaceae (A), Corynebacteriaceae (A), Cytophagaceae (B), Erysipelotrichaceae (F), Eubacteriaceae (F), Flavobacteriaceae (B), Fusobacteriaceae (Fu), Hyphomicrobiaceae (P), Methylobacteriaceae (P), Microbacteriaceae (A), Oscillatoriales (C), Oscillospiraceae (F), Pasteurellaceae (P), Peptostreptococcaceae (F), Planctomycetaceae (Pl), Propionibacteriaceae (A), Pseudomonadaceae (P), Staphylococcaceae (F), Moraxellaceae (P), Rhizobiaceae (P), Sphingomonadaceae (P), Streptomycetaceae (A), Syntrophomonadaceae (F), Thermoanaerobacterales (F), Thiotrichaceae (P), Xanthomonadaceae (P). Groups: NS, non-shifted standard chow diet; S, shifted standard chow diet; NSFS, non-shifted high-fat, high-sugar diet; SFS, shifted high-fat, high-sugar diet. Phylum: A, Actinobacteria; Ac, Acidobacteria; B, Bacteroidetes; C, Cyanobacteria; F, Firmicutes; Fu, Fusobacteria; P, Proteobacteria; Pl, Plantomycetes; T, Tenericutes; V, Verrucomicrobia. *, significant between group differences; –, not significant; (+)/(-), indicates direction of the change.

**Table 5:** Significant between group comparisons by taxon level.

Taxon Name	Diet Effect		Circadian Effect		ANOVA
	NS v NSFS	S v SFS	NS v S	NSFS v SFS	
Genus					
*Bacteroides (B)*	0.02* (−)	<0.00* (−)	–	–	<0.00*
*Parabacteroides (B)*	<0.01* (−)	–	–	–	0.01*
*Prevotella (B)*	<0.00* (−)	0.00* (−)	–	–	<0.00*
*Rikenella (B)*	–	<0.01* (−)	–	–	<0.00*
*Tannerella (B)*	<0.00* (−)	<0.00* (−)	–	–	<0.00*
*Anaerofilum (F)*	<0.00* (+)	<0.00* (+)	–	–	<0.00*
*Anaerotruncus (F)*	<0.00* (+)	–	–	–	<0.00*
*Bryantella (F)*	0.02* (−)	–	–	–	0.01*
*Catabacter (F)*	<0.00* (−)	–	0.02* (−)	–	<0.00*
*Catonella (F)*	<0.02* (−)	–	–	–	0.01*
*Clostridium-Erysipelotrichaceae (F)*	–	<0.00* (+)	–	–	<0.00*
*Clostridium (F)*	<0.00* (−)	<0.00* (−)	–	–	<0.00*
*Coprococcus (F)*	0.04* (−)	–	–	–	0.02*
*Desulfosporosinus (F)*	0.02* (+)	–	–	0.02* (−)	<0.00*
*Dorea (F)*	<0.00* (+)	–	–	–	<0.00*
*Desulfotomaculum (F)*	–	–	–	0.02* (−)	0.03*
*Enterococcus (F)*	–	–	–	–	0.03*
*Jeotgalicoccus (F)*	–	–	–	–	0.04*
*Lachnospira (F)*	0.02* (-)	–	–	–	<0.00*
*Lactobacillus (F)*	–	–	–	–	0.03*
*Roseburia (F)*	–	0.02* (−)	–	–	<0.00*
*Ruminococcus (F)*	<0.00* (+)	<0.00* (+)	–	<0.00* (+)	<0.00*
*Sporobacter (F)*	0.03* (−)	–	–	–	0.02*
*Sporosarcina (F)*	–	0.01* (+)	–	0.03* (+)	0.01*
*Tindallia (F)*	–	0.02* (+)	–	–	0.01*
*Citrobacter (P)*	0.03* (+)	–	–	–	0.02*
*Enterobacter (P)*	0.01* (+)	–	–	–	<0.00*
*Escherichia (P)*	<0.00* (+)	0.02* (+)	–	–	<0.00*
*Klebsiella (P)*	–	–	–	–	0.02*
*Shigella (P)*	–	–	–	–	0.03*
*Akkermansia (V)*	0.01* (+)	0.02* (+)	–	–	<0.00*

P values indicated. Non-significant between group differences observed for: Acetanaerobacterium (F), Acetitomaculum (F), Acetivibrio (F), Acidovorax (P), Acinetobacter (P), Adlercreutzia (A), Aerococcus (F), Afipia (P), Alkaliphilus (F), Allobaculum (F), Anaerobranca (F), Anaerofustis (F), Anaeroplasma (T), Anaerosporobacter (F), Anaerostipes (F), Anaerovirgula (F), Anaerovorax (F), Anaplasma (P), Arcicella (B), Arthromitus (F), Asaia (P), Atopobium (A), Atopostipes (F), Bacillus (F), Barnesiella (B), Blastopirellula (Pl), Blautia (F), Brevibacterium (A), Butyricicoccus (F), Butyrivibrio (F), Caldicellulosiruptor (F), Caminicella (F), Candidatus Phytoplasma (T), Catenibacterium (F), Caulobacter (P), Coprobacillus (F), Cronobacter (P), Cryobacterium (A), Corynebacterium (A), Denitrobacterium (A), Desulfitobacterium (F), Desulfonispora (F), Edaphobacter (Ac), Eggerthella (A), Enhydrobacter (P), Enterorhabdus (A), Ethanoligenens (F), Eubacterium (F), Eubacterium (Erysipelotrichaceae) (F), Epulopiscium (F), Faecalibacterium (F), Finegoldia (F), Flavobacterium (B), Flexibacter (B), Frateuria (P), Fusobacterium (Fu), Gluconobacter (P), Haemophilus (P), Hespellia (F), Howardella (F), Hydrogenoanaerobacterium (F), Johnsonella (F), Lachnobacterium (F), Klebsiella (P), Lactococcus (F), Lactonifactor (F), Leptolyngbya (C), Methylobacterium (P), Moraxella (P), Odoribacter (B), Olsenella (A), Oribacterium (F), Oscillibacter (F), Oscillospira (F), Oxobacter (F), Pantoea (P), Paraeggerthella (A), Paraprevotella (B), Parasporobacterium (F), Peptococcus (F), Peptoniphilus (P), Peptostreptococcus (F), Pirellula (Pl), Pontibacter (B), Prosthecomicrobium (P), Pseudobutyrivibrio (F), Pseudoflavonifractor (F), Porphyromonas (B), Propionibacterium (A), Proteus (P), Pseudomonas (P), Ralstonia (P), Rhizobium (P), Robinsoniella (F), Roseburia (F), Ruminococcus (F), Slackia (A), Sphingobium (P), Sphingomonas (P), Sphingopyxis (P), Sporanaerobacter (F), Sporobacterium (P), Staphylococcus (F), Streptococcus (F), Streptomyces (A), Subdoligranulum (F), Sulfobacillus (F), Swaminathania (P), Syntrophomonas (F), Thiothrix (P), Turicibacter (F), Xanthomonas (P). Groups: NS, non-shifted standard chow diet; S, shifted standard chow diet; NSFS, non-shifted high-fat, high-sugar diet; SFS, shifted high-fat, high-sugar diet. Phylum: A, Actinobacteria; B, Bacteroidetes; C, Cyanobacteria; F, Firmicutes; Fu, Fusobacteria; P, Proteobacteria; Pl, Plantomycetes; T, Tenericutes; V, Verrucomicrobia. *, significant between group differences; –, not significant; (+)/(-), indicates direction of the change.

**Table 6:** Percent average abundance of taxon at the Genus level.

Genus Name	NS N = 10	S N = 10	NSFS N = 10	SFS N = 10
*Tannerella (B)*	38.8	33.0	10.9	6.3
*Clostridium (F)*	26.6	29.0	11.9	6.4
*Turicibacter (F)*	4.0	4.4	0.7	0.0
*Eubacterium (F)*	3.3	2.8	2.5	0.9
*Lactobacillus (F)*	2.9	2.9	0.5	0.1
*Roseburia (F)*	2.8	3.5	0.8	0.4
*Coprococcus (F)*	2.5	2.1	0.1	0.1
*Atopobium (A)*	2.5	3.1	0.5	0.4
*Prevotella (B)*	2.2	2.2	0.8	0.5
*Ruminococcus (F)*	2.2	2.9	28.3	56.8
*Bacteroides (B)*	2.1	2.3	1.0	0.5
*Oscillibacter (F)*	1.4	1.5	1.4	0.1
*Bryantella (F)*	0.8	0.5	0.0	0.0
*Butyrivibrio (F)*	0.8	1.1	0.1	0.0
*Oscillospira (F)*	0.8	1.1	1.7	0.5
*Anaerostipes (F)*	0.7	2.1	0.0	0.0
*Blautia (F)*	0.7	1.5	0.1	0.2
*Rikenella (B)*	0.7	0.8	0.4	0.1
*Olsenella (A)*	0.5	0.7	0.2	0.1
*Anaeroplasma (T)*	0.3	0.1	0.0	0.0
*Lachnospira (F)*	0.3	0.3	0.0	0.0
*Pseudoflavonifractor (F)*	0.3	0.2	0.4	0.1
*Barnesiella (B)*	0.2	0.1	0.0	0.0
*Catonella (F)*	0.2	0.1	0.0	0.0
*Erysipelotrichaceae (F)*	0.2	0.1	0.7	1.0
*Desulfosporosinus (F)*	0.2	0.1	0.6	0.1
*Oribacterium (F)*	0.2	0.3	0.0	0.0
*Alkaliphilus (F)*	0.1	0.0	0.0	0.0
*Catabacter (F)*	0.1	0.0	0.0	0.0
*Denitrobacterium (A)*	0.1	0.0	0.0	0.0
*Desulfotomaculum (F)*	0.1	0.1	0.2	0.0
*Dorea (F)*	0.1	0.1	1.2	0.6
*Enterorhabdus (A)*	0.1	0.1	0.2	0.1
*Hydrogenoanaerobacterium (F)*	0.1	0.0	0.1	0.0
*Leptolyngbya (C)*	0.1	0.0	0.0	0.0
*Parabacteroides (B)*	0.1	0.0	0.0	0.0
*Paraprevotella (B)*	0.1	0.0	0.0	0.0
*Slackia (A)*	0.1	0.1	0.0	0.0
*Acetivibrio (F)*	0.0	0.0	0.1	0.0
*Akkermansia (V)*	0.0	0.0	4.9	5.2
*Allobaculum (F)*	0.0	0.0	9.5	0.0
*Anaerofilum (F)*	0.0	0.0	1.8	1.6
*Anaerotruncus (F)*	0.0	0.1	0.2	0.1
*Brevibacterium (A)*	0.0	0.0	2.7	0.7
*Butyricicoccus (F)*	0.0	0.1	0.0	0.0
*Caminicella (F)*	0.0	0.0	0.1	0.0
*Enterobacter (P)*	0.0	0.0	0.5	0.3
*Enterococcus (F)*	0.0	0.0	6.3	10.8
*Escherichia (P)*	0.0	0.0	2.4	1.5
*Faecalibacterium (F)*	0.0	0.0	0.0	0.0
*Johnsonella (F)*	0.0	0.0	0.1	0.0
*Klebsiella (P)*	0.0	0.0	0.2	0.0
*Parasporobacterium (F)*	0.0	0.1	0.0	0.0
*Propionibacterium (A)*	0.0	0.0	0.1	0.0
*Pseudobutyrivibrio (F)*	0.0	0.1	0.1	0.0
*Shigella (P)*	0.0	0.0	0.3	0.2
*Sporobacterium (F)*	0.0	0.0	0.0	0.1
*Staphylococcus (F)*	0.0	0.0	5.0	3.6
*Tindallia (F)*	0.0	0.0	0.2	0.4

Groups: NS, non-shifted standard chow diet; S, shifted standard chow diet; NSFS, non-shifted high-fat, high-sugar diet; SFS, shifted high-fat, high-sugar diet. Phylum: A, Actinobacteria; B, Bacteroidetes; C, Cyanobacteria; F, Firmicutes; T, Tenericutes; P, Proteobacteria; V, Verrucomicrobia.

**Figure 7:**
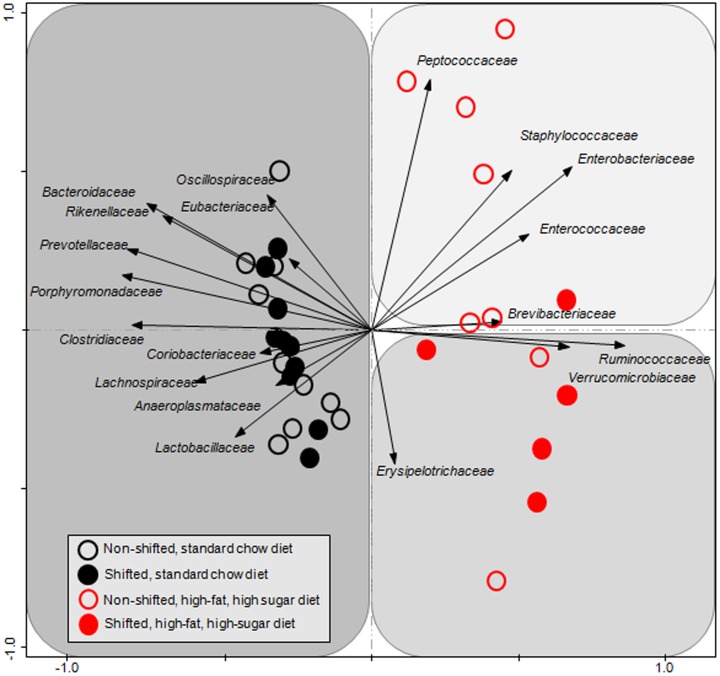
Family-level gut microbial community analysis. The composition of bacterial communities from each sample, grouped at the family level, was analyzed using principal component analysis of log-transformed and standardized data, as described in the text. Vectors, or arrows, point in the direction of the steepest increase of values for the corresponding family. The angle between arrows indicates approximated correlation (>90° indicates negative correlation). The samples are indicated with individual symbols, according to treatment, and the distance between symbols approximates the dissimilarity of their microbial communities, as measured by Euclidean distance. PCA axes 1 and 2 explain 47.51% of the variation.

**Figure 8:**
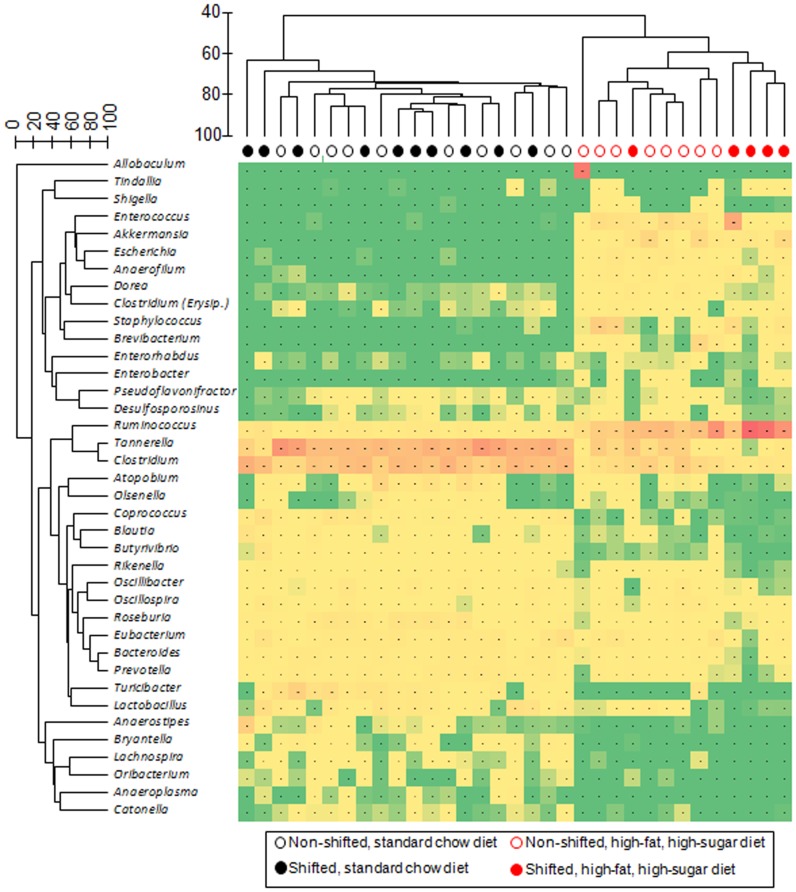
Genus-level gut microbial community analysis. A dual hierarchical dendogram describes the 40 most abundant genera detected in the amplicon sequence study (y-axis) across the mouse fecal samples. The heat map indicates the relative abundance of sequences derived from bacteria belonging to each genus, scaled to each sample (red = most abundant; green = no sequences) (x-axis). The clustering of samples was performed on the full dataset of sequences, and sequence data abundance values were standardized by sample, square-root transformed, and a resemblance matrix was generated using Bray-Curtis similarity. Similarly, hierarchical clustering was performed on the 40 most abundant species, using standardized abundance data and Bray-Curtis similarity. Group average hierarchical clustering was performed on both matrices.

Intestinal bacterial communities from standard chow-fed mice were dominated by a diverse set of bacteria from the phyla Firmicutes (e.g., families: Oscillospiraceae, Eubacteriaceae, Clostridiaceae, and Lachnospiraceae) and Bacteroidetes (e.g., families Bacteroidaceae, Rikenellaceae, Prevotellaceae, and Porphyromonadaceae) (Figure 7) with the most abundant genera of bacteria belonged to *Tannerella* and *Clostridium*. While *Tannerella* and *Clostridium* were also abundant in high-fat, high-sugar diet-fed mice, bacteria from the genus *Ruminococcus* had the highest relative abundance (Figure 8). The dominance by bacteria from the genus *Ruminococcus* was further increased in circadian rhythm disrupted, high-fat, high-sugar diet-fed mice, and is a major driver behind the observed differences between diet and circadian rhythm-disrupted intestinal microbial communities (Figures 7 and 8).

**Figure 9:**
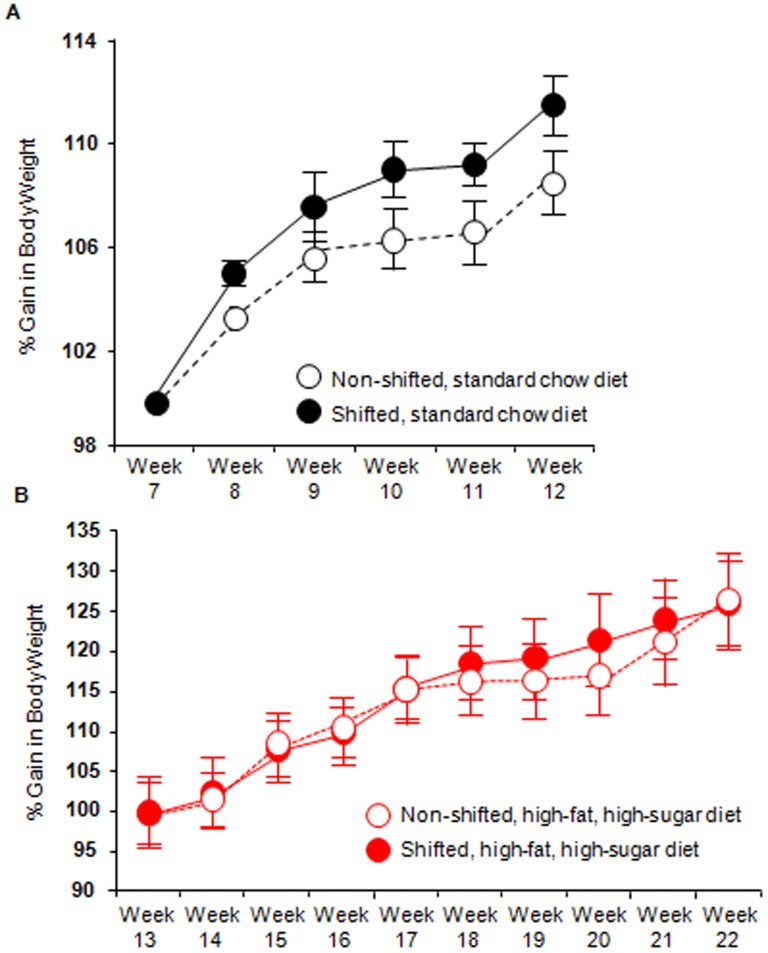
Circadian disruption has a significant effect on body weight. Mice were weighed once weekly and the body weights of mice consuming either the Standard chow diet (A) or the high-fat, high-sugar diet (B) are depicted over the last six weeks of each diet calculated as a percent of either Week 7 or Week 17, respectively. (A) Both circadian rhythm disruption (F_(1,90)_ = 4.93, p = 0.04) and time (F_(5,90)_ = 20.24, p<0.0001) had a significant impact on body weight when mice were consuming the standard chow diet. (B) There was no effect of circadian disruption on body weight in the high-fat, high-sugar diet-fed mice (F_(1,159)_ = 0.03, p = 0.85) while time did have a significant impact (F_(9,159)_ = 43.14, p<0.0001).

Obesity (human and rodent) has been associated with alterations in intestinal microbiota, including decreased average abundance of Bacteroidetes and increased Firmicutes similar to what was observed in the current study. Thus, we assessed the effects of diet and disruption of circadian organization on body weight to determine whether change in body weight could explain the changes in microbiota community structure induced by either the high-fat, high-sugar diet or as a consequence of disrupted circadian rhythms. We found a significant effect of circadian rhythm disruption (p = 0.04) as well as time (p<0.0001) in standard chow-fed mice whereby the circadian disorganized mice weighed more than the non- shifted counterparts (Figure 9A). In contrast, high-fat, high-sugar diet-fed mice showed a significant effect of time in weeks (p<0.0001) but no circadian disruption effect on body weight (Figure 9B). This effect occurred despite the fact that circadian rhythm disrupted mice consume less food than their non-shifted counterparts [6]. Thus, at a time when the intestinal microbiota community structure was statistically indistinguishable between non-shifted and shifted mice (i.e., standard chow fed mice) chronic shifting of the host circadian clock had a significant effect on body weight; while at a time when the microbiota community structure was different between non-shifted and shifted mice (i.e., high-fat, high-sugar diet-fed mice), body weight was statistically indistinguishable (Figure 9). Therefore in the current study, obesity was not a consequence of an altered microbiome and obesity does not appear to account for changes in microbiota community composition induced by the high-fat, high-sugar diet and by disruption of circadian organization.

## Discussion

It is well-established that diet impacts intestinal microbiota composition and diversity. Diets high in fat tend to decrease Bacteroides and increase Firmicutes with increases in Proteobacteria also reported [22], [23]. Similar effects are observed when a high-fat diet is combined with a high-sugar diet to comprise a “Western” diet [24], [25]. The “Western” diet-induced microbiome is distinct from that occurring as a consequence of diets high in fat or sugar [25]. The “Western” diet microbiome is dominated by Firmicutes of the Mollicute class whereas diets high in fat or sugar are comprised of Firmicutes of other classes [25]. In the current study, stool collected from mice fed a high-fat, high-sugar diet demonstrated a significant relative decrease in the abundance of Bacteroidetes and an increase in Firmicutes (i.e., non-Mollicute). *Ruminococcus* (a Firmicute of Clostridiales lineage) was particularly enriched in the stool of high-fat, high-sugar diet-fed mice. *Ruminococcus* spp. increase in response to diets high in resistant starch [26], [27] and certain species are increased in inflammatory bowel disease (i.e., ulcerative colitis, Crohn's disease) and colon cancer [28]–[30] suggesting that the increase may reflect mucolytic activity which may disrupt barrier function and cause inflammation. Likewise, *Akkermansia*, a mucolytic bacteria, increased in response to the high-fat, high-sugar diet [31]. Therefore, the diet-induced changes may have negative pro-inflammatory consequences in the intestine. It has been suggested that *Akkermansia mucinphila* has anti-inflammatory effects and improves intestinal barrier function [32]; however, in the context of our recently published paper [6] demonstrating that circadian rhythm disruption promotes intestinal hyperpermeability the likelihood of *Akkermansia* playing a beneficial role in the current study is unlikely.

Disturbing the circadian clock system negatively impacts health and it is noteworthy that repeated phase shifts only altered the intestinal microbiota composition in circadian disrupted mice fed a high-fat, high-sugar diet but not in mice that were fed the standard chow diet. Indeed, our group as well as others have demonstrated that repeated phase shifts in the light:dark cycle have minimal health effects in an "unchallenging" environment under which laboratory animals are usually maintained; however, negative consequences of chronic circadian disruption are revealed when animals are put into a “challenging” environment. For example: repeated phase shifts in the entraining light:dark cycle results in increased gut leakiness when mice are challenged with an alcohol-containing diet [6] and chronic phase shifts increase mortality and cause severe colonic inflammation only when mice are challenged with dextran sodium sulfate (DSS) [7]. Our current findings and these examples support that a second environmental insult is often necessary to reveal the deleterious effects of circadian disruption. It will be of interest to determine if other conditions that impact the microbiota community such as obesity, metabolic syndrome, inflammatory bowel disease, and stress [3], [4] have a greater effect on the microbiota environment under conditions where the circadian organization is simultaneously disrupted.

Inflammation appears to be the central mechanism of the deleterious health consequences of circadian disorganization, as exemplified by increased risk and severity of obesity, metabolic syndrome, cardiovascular disease, and cancer [33]–[35]. Since the intestinal microbiota is a trigger for systemic inflammation, we hypothesize that circadian-induced changes in microbiota composition play a role in mediating the deleterious health effects of circadian disorganization. There is significant interplay between the intestinal microbiota and intestinal epithelial cells [36] and this may be driving the intestinal hyperpermeability that we have observed [6] to promote intestinal and systemic inflammation. Although several circadian-induced effects on the microbiota were noted, the most dramatic effect was to augment the relative average abundance of the pro-inflammatory bacteria R*uminococcus* [28]–[30], suggesting that circadian-induced changes may augment mucolytic activity to disrupt intestinal barrier function and promote inflammation. In addition, it is now established that the microbiota impact intestinal epithelial barrier integrity via a downstream mechanism involving NF-ΚB [37] with members of the genus *Lactobacillus* inhibiting NF-KB [38], [39]. In the present study, relative abundance of *Lactobacillus* was decreased in the stool of high-fat, high-sugar diet-fed, circadian disrupted mice. This finding aligns with our previous studies demonstrating that circadian rhythm disruption promotes DSS-induced colitis and intestinal hyperpermeability [6], [7], both pathological states where barrier integrity is disrupted. Thus, there are multiple mechanisms by which microbiota can disrupt intestinal barrier integrity, trigger systemic inflammation, and promote pathologies (e.g., metabolic syndrome, cancer, cardiovascular disease) [40], [41].

Stress (i.e., physical, psychological) affects the intestinal microbiome in laboratory animals [42]–[45] and humans [46], [47]. For example, social disruption increases both Bacteroidetes and Clostridia (i.e., Firmicutes) whereas grid-floor-induced stress increases Bacteroidetes and Actinobacteria and physical restraint stress has been reported to reduce overall bacterial community diversity and richness [48]. Our current study distinguishes itself from these previous studies in that significant circadian-induced effects were limited to bacteria from the phylum Firmicutes. One interpretation of these data is that chronic circadian disruption is a form of long-term biological stress capable of inducing changes in microbiota that differentiates itself from other types of physical or psychological stress.

It is noteworthy that in humans, chronic circadian disruption, which occurs in shift workers, is associated with reduced sleep time and disturbance in the timing of sleep relative to other 24-hour biological rhythms [49]. Sleep loss is associated with disturbances of hormone secretion and metabolism, and voluntary partial sleep curtailment is a widespread practice in modern societies. Indeed chronic short sleep has been associated with obesity, diabetes, and cardiometabolic disorders, which themselves have been linked with the composition of the microbiota and intestinal hyperpermeability [50], [51]. For example, we have reported that disruption of sleep in mice increases the severity of symptoms in a DSS model of colitis [10]. Furthermore, sleep disruption is a feature commonly reported in patients with inflammatory bowel disease [52], [53] and a recent study found that patients with disrupted sleep have a higher risk of disease flare-up during the following six months indicating the deleterious impact of poor sleep [54]. The role of microbiota and dysbiosis in pathogenesis of colitis is now well-established [3]; thus, the negative impact of disruption of sleep and circadian rhythms on IBD course could be due to dysbiosis induced by circadian misalignment. The changes in the microbiota that we observed in the present study could be due, at least in part, to chronic sleep loss. Further studies are needed to differentiate the effects of chronic circadian disruption and chronic sleep loss as possible contributing factors to microbiota dysbiosis.

The finding that the intestinal microbiome can be altered following chronic disruption of normal circadian function raises the possibility that the composition of the microbiome at other anatomical sites (e.g., skin, vagina) might also be impacted by disturbances in the local or organism-wide circadian organization. Given the rapidly growing evidence that different microbiome structures may underlie a variety of pathological disorders, it is of great importance to determine how disruption of normal circadian time structure, such as that which occurs in the elderly, in shift workers, during jet lag, and in many individuals living on different sleep-wake schedules during the week compared to weekends and holidays (i.e., social jet lag) impacts the microbiota at various anatomical sites. Indeed, it may well be that many of the adverse effects that have been observed in circadian rhythm disrupted humans may be due in part to dysbiosis.

There are some limitations associated with or study. (1) Our study did not include age matched chow-fed mice therefore it is difficult to assess what changes may have occurred as a consequence of age. To the best of our knowledge, there are no studies demonstrating age-related effects on the microbiota but establishing this in our model will be useful to best interpret our results. (2) Likewise, the lack of an age-matched chow fed control group means it is difficult to assess the potential impact of the liquid diet itself or the content (e.g., availability of fermentable fiber/carbohydrates) on our outcomes. Future studies evaluating the effect of a high-fat diet in solid form will certainly be informative on this matter. (3) Finally, future studies evaluating microbial community function will be informative in determining the effects circadian rhythm-induced changes in microbiome function including short chain fatty acid production, metabolites linked to stress, obesity, insulin resistance, inflammation, and the gut-brain axis. Despite these limitations we have, for the first time, determined that circadian disorganization impacts the intestinal microbiome in high-fat, high-sugar fed mice.

The present results add a new environmental factor that can alter the intestinal microbiota; however, it is not clear how disruption of circadian rhythms induces changes in the intestinal microbiota community. Our hypothesis is that disrupted host circadian organization alters the circadian clock of the microbiota leading to a change in the intestinal microbiota community structure. However, at the present time, there is no evidence that the intestinal microbiota possess intrinsic circadian organization that is independent from the host circadian structure although it has been established that cyanobacteria harbors a circadian timing mechanism. It has been conservatively estimated that animals have had a symbiotic relationship with microbes for 500 million years, and given the recent evidence that circadian clocks may have evolved in prokaryotes 2.5 billion years ago [55], [56], the host and microbiota circadian clock systems may have co-evolved with nutrient availability and/or the type of nutrient composition in the diet acting to entrain circadian rhythms in the intestinal microbiota. Our findings provide a rationale to study intrinsic circadian clock in intestinal microbiota and the potential impact of host circadian organization on the bacterial circadian clock system.
